# Perineural Release with or Without Balloon Decompression for the Treatment of Lumbosacral Discogenic Syndromes in Professional Athletes

**DOI:** 10.3390/neurosci7050103

**Published:** 2026-09-19

**Authors:** Yasin Said AlMakadma, Jouhara Jouhar, Abdulaziz Farooq, Karim Chamari, Tahani AlBatarni

**Affiliations:** 1 Interventional Pain and Neuromodulation, Aspetar Orthopaedic and Sports Medicine Hospital, Doha 29222, Qatar; jouhara.jouhar@aspetar.com (J.J.); tahani.albatarni@aspetar.com (T.A.); 2 Faculty of Medicine, Qatar University, Doha 29222, Qatar; 3 Medical College, Weil Cornell Medical College-Qatar, Doha 29222, Qatar; 4 Research and Scientific Support Department, Aspetar Orthopaedic and Sports Medicine Hospital, Doha 29222, Qatar; 5 Naufar Center, Doha 29222, Qatar; 6 Higher Institute of Sport and Physical Education, ISSEP Ksar Saïd, Manouba University, Tunis 2010, Tunisia

**Keywords:** lumbosacral discogenic syndrome, perineural release, balloon decompression neuroplasty, patient-reported outcome measures, low back pain, perineural release

## Abstract

**Background:** Lumbosacral discogenic syndrome (LSDS) is a common cause of low back pain (LBP) and lower limb pain (LLP) in athletes, often resulting in impaired sporting performance and delayed return to sport (RTS). Evidence regarding minimally invasive interventions, such as epidural perineural release (PNR), remains limited in athletic populations. **Aim:** This study aimed to compare outcomes following PNR with versus without balloon decompression neuroplasty in athletes with LSDS. **Methods:** We conducted a retrospective review of PNR performed between 2019 and 2024 for the treatment of LSDS among professional athletes. Demographic, clinical, and outcome data were collected from medical records and follow-up assessments. Pain intensity was assessed using the Numerical Rating Scale (NRS), while patient-reported outcomes were evaluated using the EuroQoL-5D (EQ-5D). Additional outcomes included pain-free walking, standing, and sitting durations, and self-reported health scores. **Results:** A total of 33 patients who had one intervention were included. Significant improvements were observed in all pain, functional, and quality-of-life outcomes following treatment (all *p* < 0.001). Maximum, minimum, and average pain intensity decreased from 7.5 ± 1.7 to 2.8 ± 2.2, 3.8 ± 2.3 to 0.3 ± 0.7, and 5.6 ± 1.7 to 1.5 ± 1.2, respectively. All EQ-5D domains and quality of sleep also improved significantly (all *p* < 0.001). Pain-free walking, standing, and sitting durations increased significantly, from 24.8 ± 23.2 to 49.7 ± 26.7 min, 18.4 ± 20.0 to 44.1 ± 23.1 min, and 25.5 ± 23.7 to 44.6 ± 24.9 min, respectively (all *p* < 0.001). Self-reported health scores improved from 51.9 ± 22.6 to 83.0 ± 14.3 (*p* < 0.001). **Conclusions:** Epidural perineural release, with or without balloon decompression neuroplasty, was associated by significant improvements in pain, functional capacity, and quality of life among athletes with LSDS refractory to conservative management. These findings suggest that PNR may be a promising minimally invasive treatment option; however, controlled prospective studies are required to establish its effectiveness and determine the additional benefit of balloon decompression.

## 1. Introduction

Professional athletes are susceptible to various musculoskeletal injuries and degenerative conditions affecting the knee joints, tendons, and spine [1,2,3,4]. Among these, spinal injuries and diseases are particularly prevalent and occur more frequently in athletes than in the general population [5,6]. Lumbosacral discogenic syndrome (LSDS), which causes low back pain (LBP) and/or lower limb pain (LLP), can have a prolonged detrimental effect on professional athletes [6,7,8]. LSDS is associated with decreased sporting performance and may result in delays or an inability to return to sport (RTS) [9]. Both acute and chronic discopathies are more common in athletes than in the general population, with certain sports—such as weightlifting, gymnastics, mogul skiing, and football—posing a higher risk [6,10,11]. Vertebral ring fractures and spondylolysis are especially prevalent among adolescent athletes and typically present with persistent LBP [5,12].

The management of discogenic syndromes in athletes requires a thorough assessment, accurate diagnosis, and careful consideration of the implications for the athlete’s career and return to sport (RTS). The choice between surgical and less invasive treatments should be made cautiously [9]. While surgical approaches can be effective, they have inherent drawbacks and do not guarantee a full or prompt RTS [9]. There is limited data available to guide clinicians in managing lumbar spine discogenic syndromes (LSDS) and to assist sports physicians in deciding between surgical and non-surgical options [9,13]. Interventional therapies, ranging from steroid injections to advanced techniques such as perineural release (PNR), also known as epidural adhesiolysis, may offer less invasive alternatives with shorter recovery times compared to surgery [14].

### PNR Techniques

**Figure 1:**
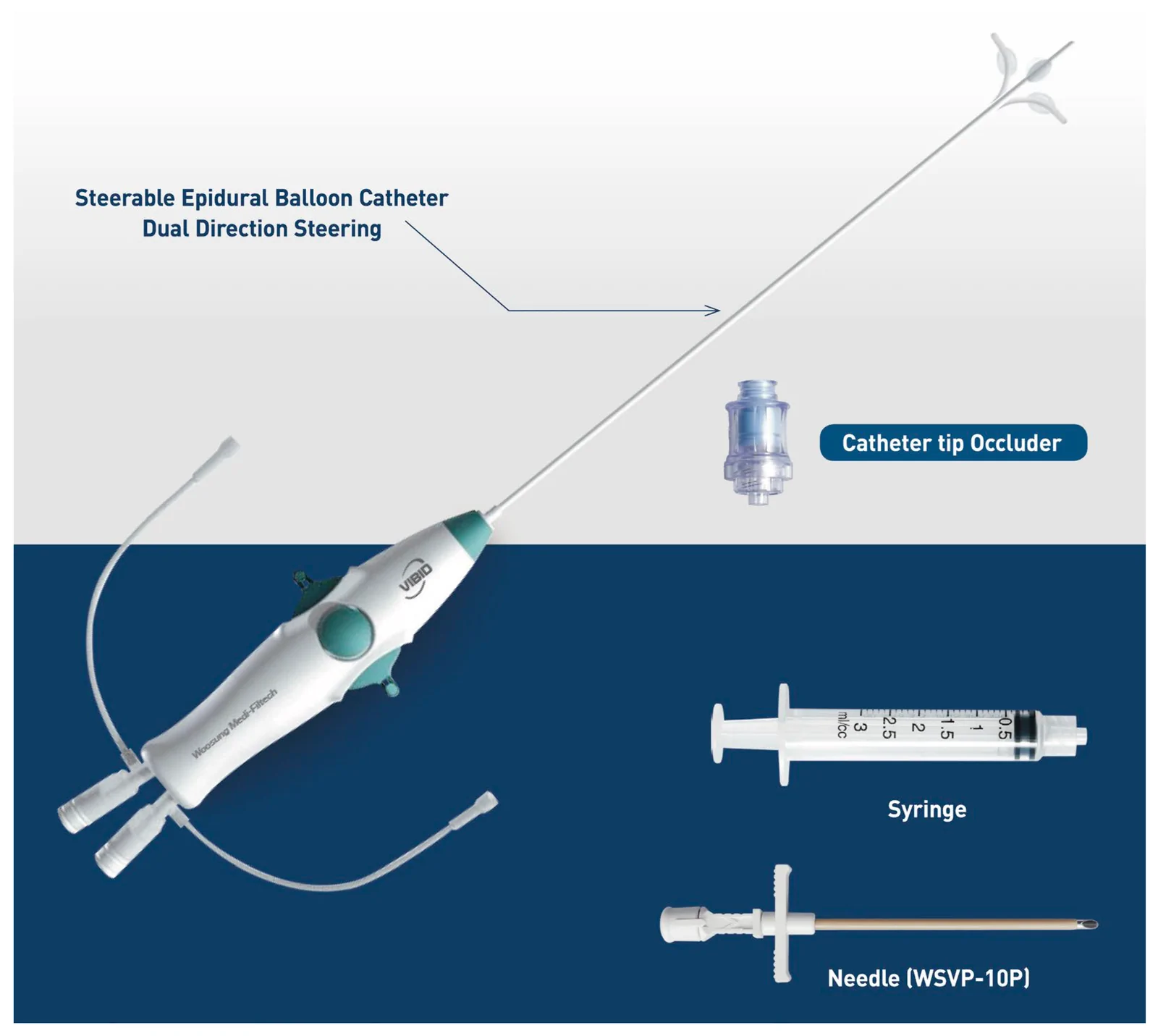
VIBID steerable catheter devices (Woosung Medi-Filtech; https://woosungmt.co.kr/images/pdf/VIBID.pdf (accessed on 1 September 2026).

PNR techniques are performed aseptically in an operating room setting under antibiotic prophylaxis. Access to the epidural space is obtained through the sacral hiatus after administering local anesthetics and adequate sedation and analgesia provided by a senior anesthetist. The procedure is guided by fluoroscopy, with the patient in a prone position and pressure points protected. Various steerable tools are available for PNR, including options or without or with a balloon tip for PNRBDC (Figure 1). Once access to the caudal epidural space is achieved, confirmation is obtained by injecting 1 to 2 milliliters of contrast media (Iohexol 300).

**Figure 2:**
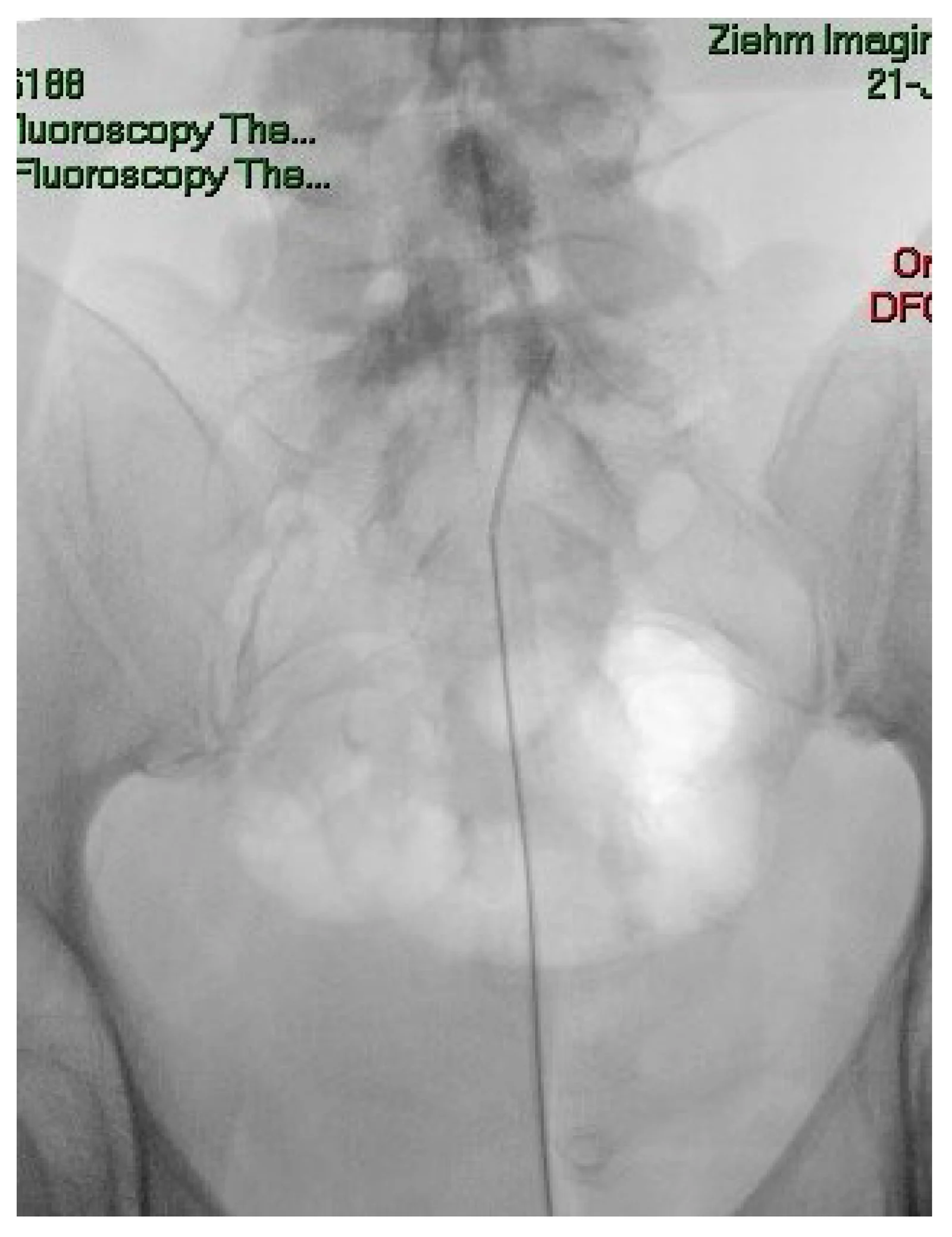
Preoperative radiculography.

The steerable tip, in the next step, is placed at the level of the second sacral and third sacral vertebrae, and further media contrast is injected to identify “filling defects” in the perineural medium, indicating the presence of compression and/or adhesive tissue (Figure 2).

**Figure 3:**
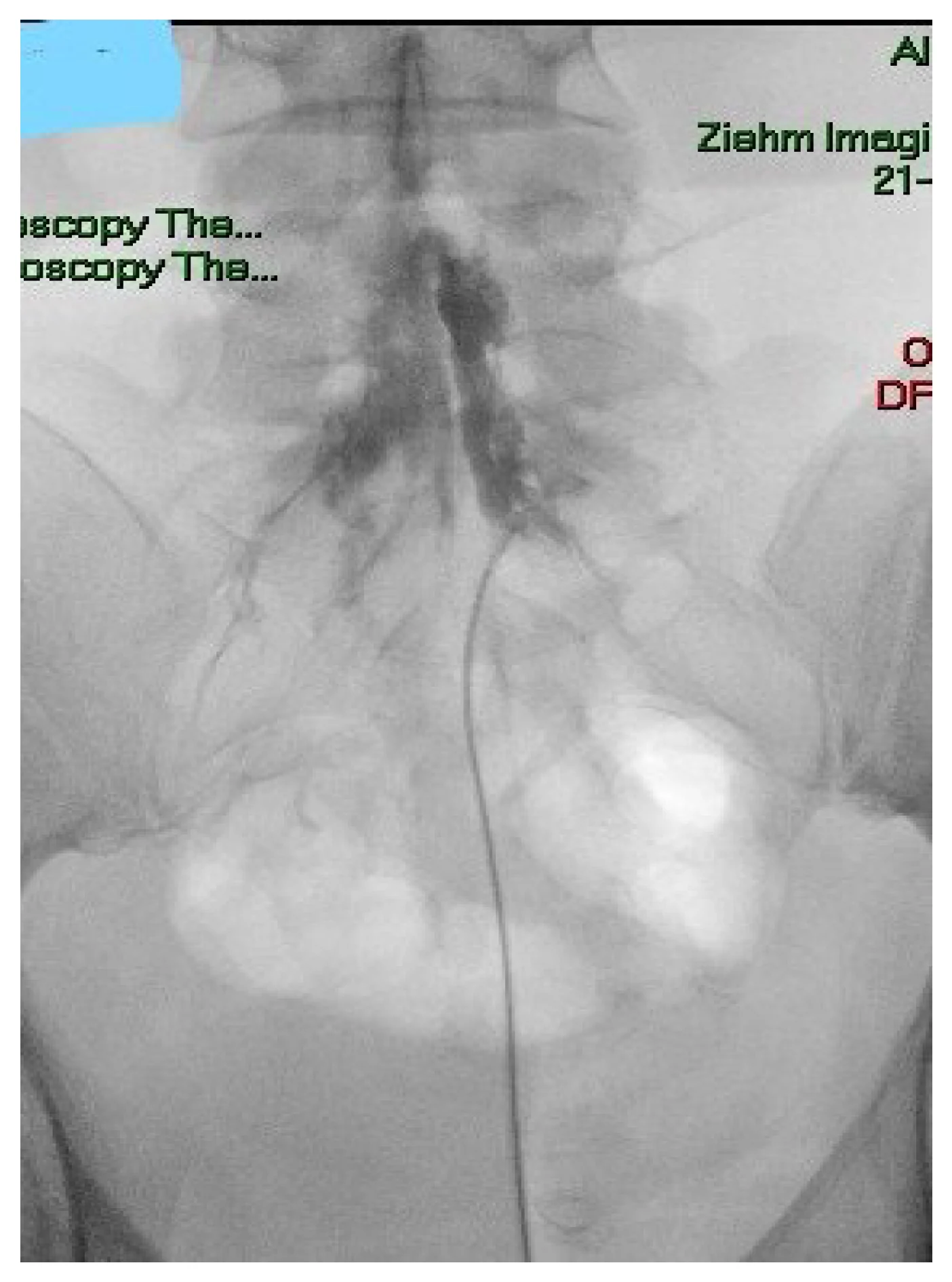
Radiculography after completion of PNR.

The steerable tip is then guided to defect areas and boluses (2–5 mLs) of 3% hypertonic saline are slowly injected through 10 mL syringes, alternating with 300 IntUnit of hyaluronidase. The tip should now gently be pushed to go through the defect. In cases where this fails, balloon decompression is carefully performed—inflation lasts <10 s and is repeated after deflation of the balloon. At the end of the process, a mixture of 8 mg of Dexamathasone and 3 mL of LevoBupivacaine 0.5% (total volume 5 mLs) is injected and flushed with 2–3 mL of normal Saline. Figure 3 shows the improvement (resolution) of filling defects at the end of the procedure.

The patient is then turned supine and taken to the post-anesthetic care unit, where routine vital monitoring is continued. The patient returns to the ward for a few hours’ stay before being discharged home.

## 2. Materials and Methods

### 2.1. Study Design

This retrospective cohort study analyzed data from athletes who underwent epidural perineural release (PNR), with or without balloon decompression neuroplasty (PNRBDC), for lumbosacral discogenic syndrome (LSDS) between January 2019 and December 2024 and was performed at the Orthopedics and Sports Medicine Hospital of Aspetar. Outcomes in terms of the Numerical Rating Scale (NRS), EQ-5DS, pain-free walking, standing and sitting duration, self-reported health scores, and return-to-sport (RTS) rates were compared between the pre- and postoperative statuses. The study reports data from the athlete subgroup of a previously published retrospective cohort study.

### 2.2. Setting

The study was conducted at the Aspetar Orthopedic and Sports Medicine Hospital, Doha, Qatar. Eligible patients were identified through operating room records. Clinical data was obtained from routinely documented electronic medical records and supplemented with outpatient follow-up visits and telephone assessments when required.

### 2.3. Participants

Operating room records were reviewed to identify all registered athletes who underwent a primary PNR, with or without balloon decompression neuroplasty, for LSDS between 2019 and 2024.

Athletes are defined and registered as such at our institution if they are professional athletes actively practicing their sports and linked to international, regional, or national clubs and/or the Qatar Olympic Committee as well as military teams when applicable.

Athletes aged 18–50 years with clinically and radiologically confirmed discogenic syndrome were included if they had persistent symptoms despite an adequate trial of conservative management, including pharmacological therapy and other nonoperative treatments. Patients were required to have experienced the current symptomatic episode for at least 14 days before intervention and to have available data from at least one in-clinic follow-up for a minimum of 14 days after the procedure. Routine practice includes patient history and symptoms of pain and paresthesia; clinical findings of sensory alteration (pinprick, touch, and positive SLR); and consistent discopathy/stenosis findings in the MRI.

Although a 30-day symptom-duration threshold might provide a more stringent definition of persistent symptoms, we selected a minimum duration of 14 days to reflect the clinical context of patients with refractory discogenic pain who had already failed conservative management. This approach avoided unnecessarily delaying intervention in patients with persistent, functionally limiting symptoms despite appropriate nonoperative treatment. Within our cohort, only one patient had reported 15 days of continuously worsening symptoms prior to the procedure. Importantly, this patient had a history of intermittent, relapsing symptoms over several years and months and had experienced a sustained increase in symptom severity leading to the intervention and was included.

### 2.4. Procedures

#### 2.4.1. Indications

PNR, either alone or combined with balloon decompression neuroplasty, was offered to athletes with persistent low back pain with or without lower limb pain secondary to LSDS when conservative management had failed and clinical findings were consistent with radiological imaging. Balloon decompression neuroplasty was performed when epidural adhesions or technical difficulty in catheter advancement through the epidural space were anticipated or encountered during the procedure.

All patients underwent comprehensive clinical assessments, neurological examinations, and imaging review before intervention. Procedures were performed under conscious sedation and local anesthesia in the prone position using strict aseptic technique following prophylactic intravenous antibiotics according to institutional protocol.

#### 2.4.2. Epidural Perineural Release Procedure

All procedures (PNR or PNRBDC) were performed by the same senior consultant specializing in interventional pain management and neuromodulation. The choice of technique (PNR or BNRBDC) depended on multiple factors such as the number of affected intervertebral disks and the expected difficulty of the procedure caused by stenosis and the chronicity of the discopathy.

### 2.5. Outcome Measures

Pain intensity was assessed using the 11-point Numerical Rating Scale (NRS: 0 for no pain; 10 for the worst imaginable pain), including maximum, minimum, and average pain intensities.

Health-related quality of life was assessed using the EuroQol Five-Dimension questionnaire (EQ-5D), evaluating mobility, self-care, usual activities, pain/discomfort, and anxiety/depression. Each dimension was scored on a five-level ordinal scale (1 to 5) ranging from no problems (1) to extreme problems (5).

Additional patient-reported outcomes included sleep quality, pain-free walking, standing and sitting duration, self-reported overall health score (0–100%), and return-to-sport (RTS) status where available. RTS was defined as being able to return to training (at the athlete’s club) and join competitions with the team when applicable. No specific monitoring was performed for partial returns. The decision to allow RTS was taken in conjunction with physiotherapy team and at the time when the patient’s symptoms were resolved. The effective date for RTS was retained based on the above definition.

### 2.6. Bias

To minimize selection bias, all eligible athletes identified during the study period were included, and patients who had other procedures (other than PNR or PNRBDC) performed during the same episode were excluded. Data extraction followed standardized procedures using electronic medical records and follow-up documentation.

### 2.7. Follow-Up Assessments

This study analyzed retrospectively collected follow-up data. All patients had at least one in-clinic follow-up which was carried out 29 days or more post-operatively.

When athletes had multiple follow-up assessments, the most recent available evaluation was used for analysis to reflect the longest available clinical outcome. Additional follow-ups were performed over the phone to complete missing information, in particular to confirm the date of RTS. Data were initially recorded on standardized paper forms and subsequently entered anonymously into a dedicated LibreOffice database.

### 2.8. Confounding

The decision to perform balloon decompression neuroplasty in addition to PNR was based on routine clinical judgment according to intraoperative findings and imaging characteristics, particularly when epidural adhesions or difficult catheter advancement were encountered. Consequently, outcomes were reported separately for athletes undergoing PNR alone and those receiving PNRBDC.

### 2.9. Statistical Analysis

Statistical analyses were performed using SPSS version 21.0 (IBM Corp., Armonk, NY, USA). Continuous variables were summarized as mean ± standard deviation (SD) or median and interquartile range (IQR), depending on data distribution, while categorical variables were presented as frequencies and percentages.

Normality was assessed using the Shapiro–Wilk test. Pre- and post-intervention continuous outcomes were compared using paired parametric *t*-test or non-parametric tests as appropriate. Ordinal EQ-5D domain scores were analyzed using the Wilcoxon signed-rank test. McNemar’s test was used to compare categorical outcomes where applicable. A two-sided *p*-value < 0.05 was considered statistically significant.

### 2.10. Ethical Considerations

The study received approval from the Aspetar Institutional Review Board (IRB approval number: E202401069). As this was a retrospective study, the requirement for individual study-specific informed consent was waived. All patients were adults aged 18 and above and had previously provided written institutional general consent allowing the anonymous use of clinical data for research purposes, with the option to opt out. Patient confidentiality was maintained through complete anonymization of all collected data.

## 3. Results

### 3.1. Demographics

**Figure 4:**
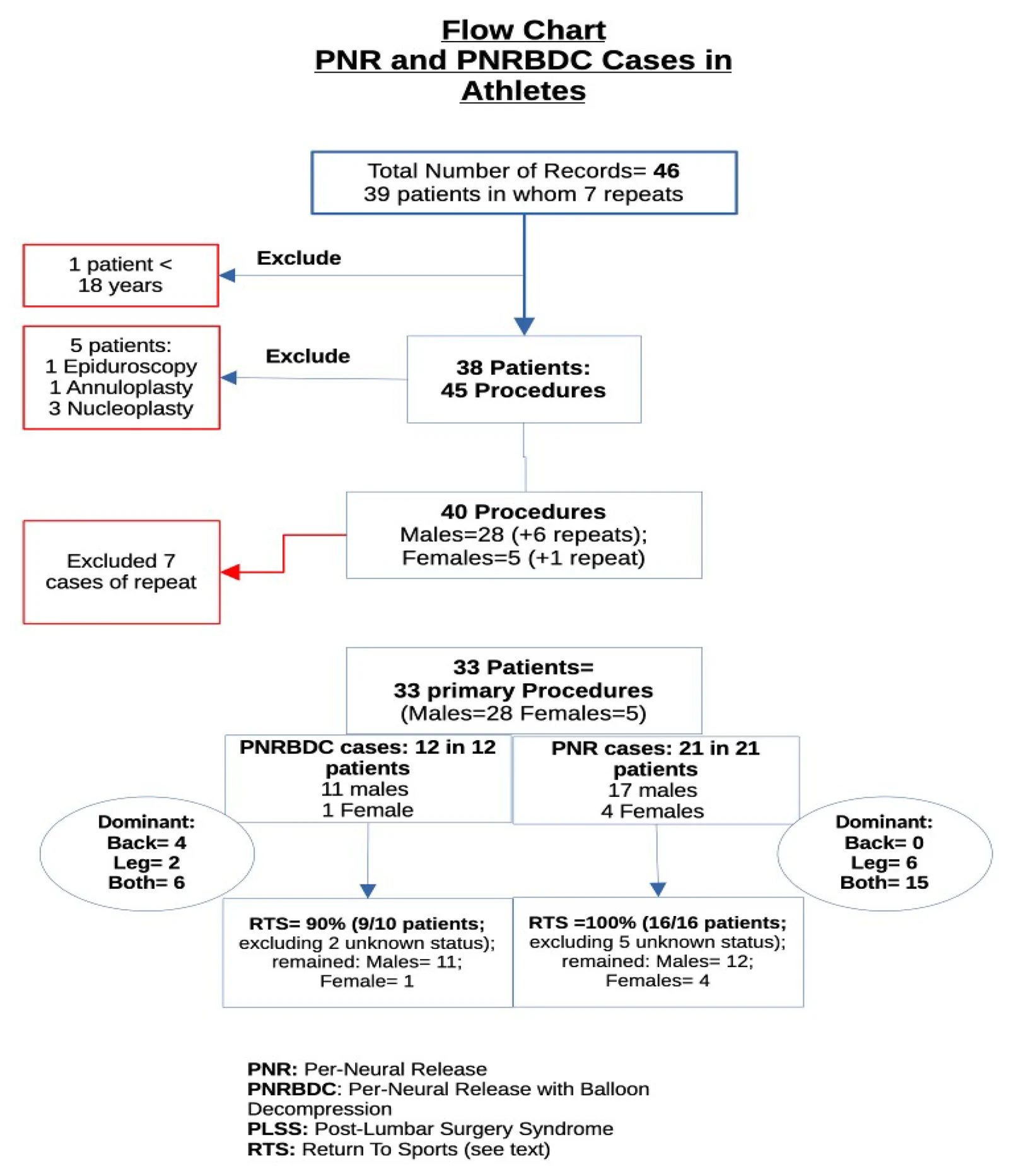
Patient flowchart.

Thirty-three athlete patients who underwent one intervention, PNR or PNRBDC, were included in the study (Figure 4). Twenty-one patients underwent perineural release (PNR) alone, while 12 others underwent PNRBDC. The mean age was 29.5 ± 7.2 years in the PNR group and 29.1 ± 8.0 years in the PNRBDC group. The cohort was predominantly male, with 17 males (81.0%) and four females (19.0%) in the PNR group and 11 males (91.7%) and one female (8.3%) in the PNR with balloon decompression group.

Most patients had undergone at least one previous minor intervention, including nerve root injections, before PNR (81.0% in the PNR group and 75.0% in the PNRBDC group). All patients tried variable modalities of conservative therapy, including physiotherapy and pharmacotherapy. The mean duration of symptoms before intervention was 21.1 ± 23.6 months in the PNR group and 20.8 ± 26.5 months in the PNR with balloon decompression group.

The interval between intervention and follow-up assessment varied across patients. In the PNR group, 38.1% were assessed within 30 days, 38.1% between 30 and <90 days, 4.8% between 90 and <120 days, 9.5% between 120 and <360 days, and 9.5% after 360 days. In the PNRBDC group, 16.7% were assessed within 30 days, 41.7% between 30 and <90 days, 8.3% between 90 and <120 days, 8.3% between 120 and <360 days, and 25.0% after 360 days (Table 1).

### 3.2. Outcome of EQ-5D, Pain, and Duration of Pain-Free Activity Pre- and Post-PNR and PNRBDC

Following PNR and PNRBDC, significant improvements were observed across all assessed outcomes (all *p* < 0.001). EQ-5D scores improved significantly across all domains, including mobility, usual activities, self-care, pain/discomfort, and anxiety/depression. Quality of sleep also improved significantly from 2.3 ± 1.2 to 1.1 ± 0.3 (*p* < 0.001). While unequal follow-up times limit the interpretation of this data, the follow-up duration was not associated with change in active living, self-care, pain, and QoS but only with mobility, as can be seen from Table 2.

Pain intensity decreased significantly following the intervention. Maximum pain intensity decreased from 7.5 ± 1.7 to 2.8 ± 2.2, minimum pain intensity from 3.8 ± 2.3 to 0.3 ± 0.7, and average pain intensity from 5.6 ± 1.7 to 1.5 ± 1.2 (all *p* < 0.001).

Functional capacity also improved significantly. Pain-free walking duration increased from 24.8 ± 23.2 to 49.7 ± 26.7 min, standing duration from 18.4 ± 20.0 to 44.1 ± 23.1 min, and sitting duration from 25.5 ± 23.7 to 44.6 ± 24.9 min (all *p* < 0.001). The self-reported health score improved from 51.9 ± 22.6 to 83.0 ± 14.3 (*p* < 0.001), indicating substantial improvement in overall health status following the intervention (Table 3).

### 3.3. Outcome of EQ-5D, Pain and Duration of Pain-Free Activity Pre- and Post-PNR

A total of 21 patients with available pre- and post-treatment data were analyzed following perineural release (PNR). Significant improvements were observed across all EQ-5D domains, including mobility, usual activities, self-care, pain/discomfort, anxiety/depression, and quality of sleep (all *p* < 0.001).

Pain intensity also decreased significantly following PNR. Maximum pain intensity decreased from 8.0 ± 1.4 to 2.7 ± 2.2, minimum pain intensity from 4.4 ± 2.0 to 0.4 ± 0.7, and average pain intensity from 6.2 ± 1.4 to 1.5 ± 1.2 (all *p* < 0.001).

Significant improvements were also observed in pain-free functional capacity. Maximum pain-free walking time increased from 21.5 ± 20.9 to 51.0 ± 15.5 min, standing time increased from 14.3 ± 17.1 to 44.0 ± 19.7 min; and sitting time increased from 19.3 ± 21.8 to 46.7 ± 26.2 min (all *p* < 0.001). The self-reported health score also improved significantly, increasing from 49.3 ± 23.0 to 84.8 ± 14.4 (*p* < 0.001). Overall, these findings demonstrate significant improvements in pain, functional capacity, quality of life, and perceived health following PNR (Table 4).

### 3.4. Outcome of EQ-5D, Pain and Duration of Pain-Free Activity Pre- and Post- PNRBDC

A total of 12 patients underwent perineural release (PNR) with balloon decompression neuroplasty. Significant improvements were observed across all EQ-5D domains, including mobility, usual activities, self-care, pain/discomfort, anxiety/depression, and quality of sleep (*p* = 0.006–0.034).

Pain intensity also decreased significantly following PNRBDC. Maximum pain intensity decreased from 6.5 ± 1.8 to 2.9 ± 2.3; minimum pain intensity from 2.7 ± 2.5 to 0.3 ± 0.7; and average pain intensity from 4.6 ± 1.7 to 1.6 ± 1.3 (*p* = 0.004, 0.011, and 0.002, respectively).

Functional outcomes showed a significant improvement in maximum pain-free standing time, which increased from 25.4 ± 23.3 to 44.2 ± 29.1 min (*p* = 0.011). Pain-free walking time increased from 30.8 ± 26.7 to 47.5 ± 40.4 min, but this change did not reach statistical significance (*p* = 0.058). Similarly, pain-free sitting time increased from 36.3 ± 23.9 to 40.8 ± 23.0 min, without statistical significance (*p* = 0.344). The self-reported health score improved significantly from 56.3 ± 22.3 to 80.0 ± 14.3 (*p* = 0.026). Overall, PNRBDC was associated with significant improvements in pain, quality of life, standing capacity, and perceived health status (Table 5).

### 3.5. Change in EQ-5D in All Patients

Across all 33 patients, substantial improvements were observed across all EQ-5D domains following PNR procedures. The proportion of patients with none-to-mild impairment increased from 42.4% to 87.9% for mobility, from 15.2% to 84.8% for activities of daily living (ADLs), and from 45.5% to 87.9% for self-care. Similarly, the proportion reporting none-to-mild pain and discomfort increased from 6.1% before the intervention to 81.8% after the intervention. Anxiety/depression also improved, with the proportion of patients reporting none-to-mild symptoms increasing from 27.3% to 87.9%. Quality of sleep showed a marked improvement, with the proportion reporting none-to-mild impairment increasing from 45.5% to 97.0%.

Overall, these findings demonstrate a substantial shift toward lower levels of impairment across all assessed EQ-5D domains following the intervention, with the greatest improvements observed in pain and discomfort, activities of daily living, anxiety/depression, and quality of sleep (Table 6).

### 3.6. Duration of Symptoms Prior to Procedures (EQ-5D)

**Figure 5:**
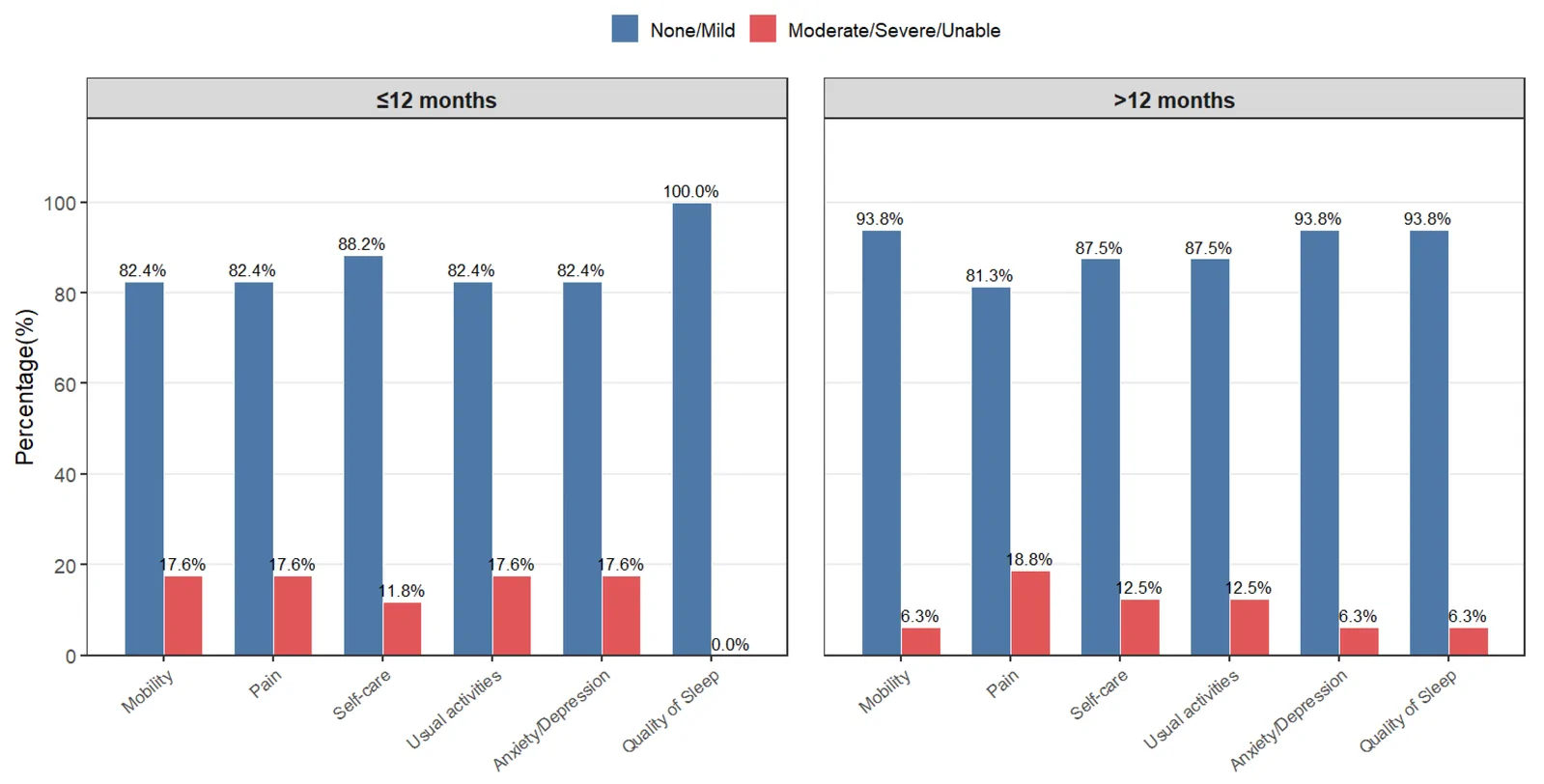
Prevalence of EQ-5D outcomes at the last follow-up, stratified by symptom duration (≤12 months and >12 months).

Among athletes with symptoms lasting either less than one year or more than one year, the interventions were associated with a statistically significant reduction in the proportion reporting severe or disabling EQ-5D impact (Figure 5). At the last follow-up, most participants in both duration groups reported no or only mild problems across all assessed domains. In the ≤12-month group, the prevalence of moderate/severe impairment ranged from 0.0% for quality of sleep to 17.6% for mobility, pain, usual activities, and anxiety/depression, with 11.8% reporting moderate/severe problems in self-care. In the >12-month group, the proportion reporting moderate/severe impairment ranged from 6.3% to 18.8%, with the highest prevalence observed for pain (18.8%), followed by self-care and usual activities (12.5% each), while mobility, anxiety/depression, and quality of sleep were affected in only 6.3% of participants. Overall, more than 80% of athletes in both symptom-duration groups reported no or mild limitations in all EQ-5D domains at the final assessment, indicating substantial improvement irrespective of symptom duration.

### 3.7. Athletes Return to Sport After the PNR Interventions

**Figure 6:**
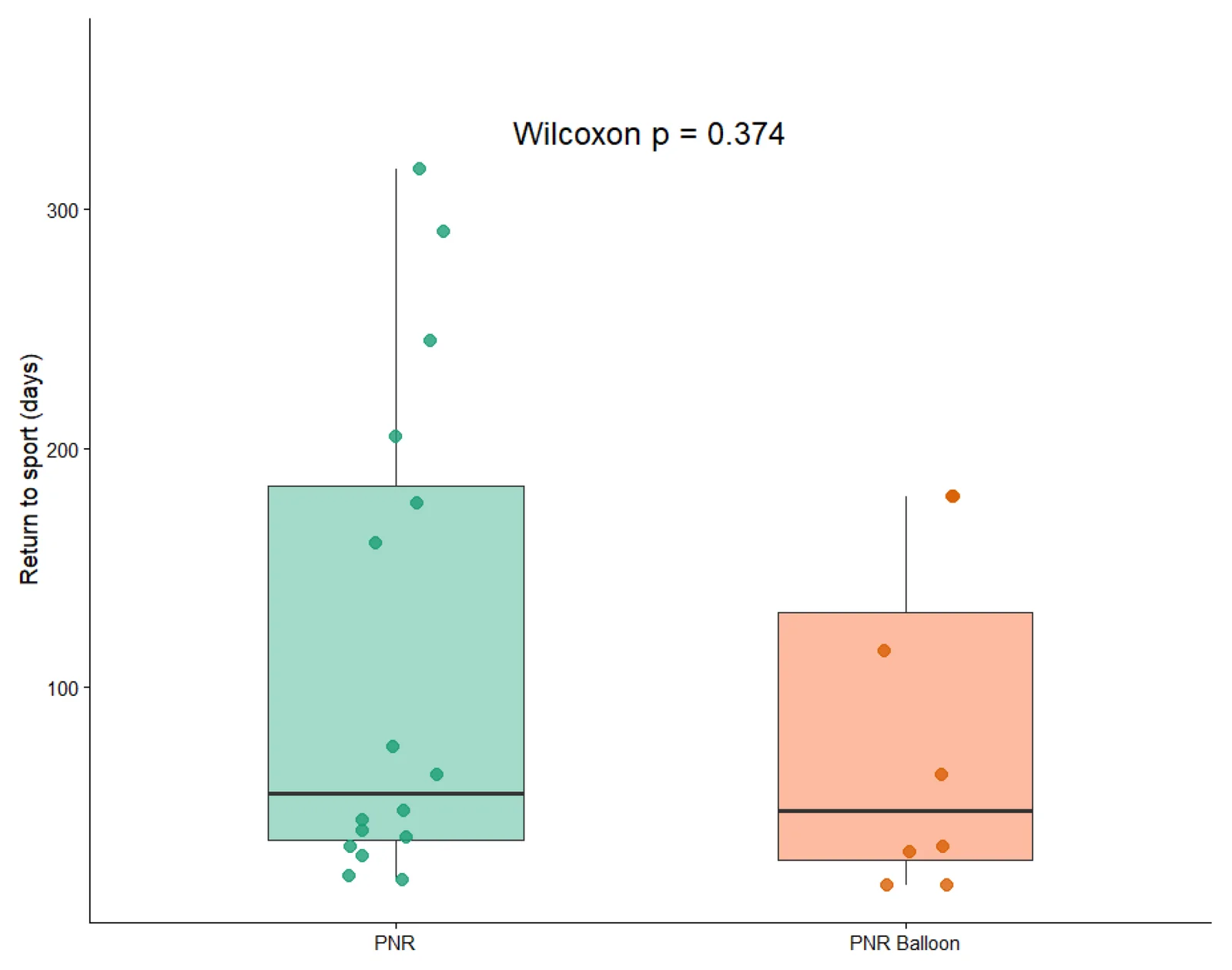
Patients RTS after PNR and PNRBDC interventions (*n* = 256).

RTS status was known in 26 patients in total, of whom one failed to RTS (in the group of PNRBDC). After excluding players whose RTS status remained unknown (*n* = 7) because they could not be contacted, out of the remaining, the valid RTS rate was 96.2% in both groups. In the PNR group, RTS status was known in 16 patients (after excluding five cases of unknown status) and was achieved in all of them (100%). In the PNRBDC group, two cases were of unknown status; in the remaining 10, nine patients had confirmed RTS (90%). When participants with unknown RTS status were included, the RTS rate was 76.2% (16/21) in the PNR group and 75.0% (9/12) in the PNRBDC neuroplasty group. Overall RTS, when patients with unknown status were included, is thus 75.8% (25/33). Among patients with available time-to-RTS data, the mean time to RTS was 113 ± 103 days in the PNR group and 80 ± 70 days in the PNRBDC group. Given the skewed distribution, the median (IQR) time to RTS was 56 (35–191) days for PNR and 48 (24–148) days for PNRBDC. There was no statistically significant difference in time to RTS between the two groups using the Wilcoxon rank-sum test (*p* = 0.374; Figure 6).

## 4. Discussion

Discopathy and LSDS are more commonly encountered in athletes than non-athlete young adults (~73% versus ~25%) [1,7]. LSDS does not only cause pain and compromise the RTS per se, but also, by its associations with other injuries, causes pain in the lower limbs in particular, and therefore represents a real challenge of management [3]. Despite its impact on the careers of professional athletes, no common consensus is available to clearly guide the management of LSDS in athletes. Relatively few reports and limited literature are available to guide the management of this important issue in athletes [9].

The outcome from purely conservative management (rehabilitation and analgesics) may not be inferior to that observed after surgical interventions that may result in surgical complications in ~35% of cases, as reported by Sedrak et al. [15]. No significant difference was found between surgical and conservative management in terms of RTS [16,17]. Nevertheless, RTS after surgery for LSDS was reported to be at lower performance levels compared to pre-surgical levels and resulted in a shortened postoperative career [15,17,18]. There is, however, some uncertainty surrounding these reports due to varying methodologies, heterogeneity of patients, low numbers of reported cases, and variation in surgical indications and probably techniques. It was also proposed that combining percutaneous endoscopic discectomy with radiofrequency thermal annuloplasty allowed all patients to return to full sport performance [19].

Similarly to that of surgery, a review of the literature reveals only a few reports on minimally invasive interventions for professional athletes diagnosed with LSDS. Jackson et al. reported a 44% improvement in athletes who received epidural selective injections for lumbar discogenic symptoms, a lower percentage than observed in the general population for the same intervention [20]. Such an outcome may indicate insufficient benefit from steroid injections alone in the treatment of LSDS or an insufficiently effective technique, probably due to limited diffusion of steroids around the diseased area. The use of PNR in our previously published general cohort was associated with significant improvement in LSDS amongst the 221 cases reported and to a better extent than reported in the literature for epidural steroid injection or conventional adhesiolysis [21].

To be more specific, therefore, we performed the present study as a sub-analysis of PNR and PNRBDC in the athlete cohort. PNR and PNRBDC were both associated with improvement of symptoms of LSDS in athletes. We observed a significant improvement in the NRS scores in the postoperative period, with the worst pain score decreasing by approximately threefold (from a mean of 7.5 to 2.8; *p* < 0.001; Table 3). This trend was consistent with self-reports (EQ-5D). Thus, prior to PNR/PNRBDC, athlete patients described their pain as severe or extreme in most cases (mean 3.8 on the scale 1–5), translating the heavy burden against athletes’ careers. Following PNR, there was a remarkable improvement in pain levels, shifting towards mild-to-no pain, with an average of 2 (mild pain; *p* < 0.001; Table 3). All other aspects of PROMs showed positive progress following the intervention.

Pain and anxiety–depression adversely influence the recovery of athletes and their RTS [22,23]. There is a complex association between the emotional and cognitive status of athletes on the one hand and the risk of injury, efficacy of rehabilitation, pattern of recovery, and RTS on the other hand [22,23]. This interaction involves fears of pain, failure to achieve, and low self-esteem and is likely to cause delayed recovery and RTS [24,25]. In our cohort, PNR/PNRBDC were associated with a meaningful improvement of anxiety–depression scores (from 3.2 ± 1 pre-procedure to 1.1 ± 0.3 post-procedure, *p* < 0.001; Table 3). Similarly, the RTS rate was, overall, 96.2% (among those with known status, 26 patients). RTS among patients who had PNR was 100% and was 90% among those who had PNRBDC (Figure 4). While we accept the limitations of our study, this high level of RTS may be valuable in considering the PNR interventions as an effective option for the management of LSDS. In addition, the observations of improved pain scores, QoS, and pain-free activities may all be positive contributors to the associated reduction in anxiety–depression scores, which is likely to encourage the RTS, a finding that could support the importance of QoS in athletes’ performance [26].

In our patients, RTS was achieved at 113 ± 103 days in the PNR group and at 80 ± 70 days in the PNRBDC group. There was no statistically significant difference (*p* = 0.374; Figure 5), with medians of 56 and 48 days, respectively. Overley et al., in their systematic review, found an average of ~ 83.5% of RTP in athletes who underwent one-level microdiscectomy but found no difference between operative and nonoperative studies [17]. Sedrak et al., in another systematic review, concluded the same and reported RTP by >155 days on average after lumbar discectomy and over 123 days in nonoperative cases; the average of RTP itself did not significantly differ [15]. We believe, while being mindful of our study limitations, that both PNR, either alone or in combination with more specific techniques like balloon decompression neuroplasty, may bridge the gap between conservative and surgical approaches in a significant number of athletes suffering from LSDS. If an athlete is unable to return to sport due to LSDS symptoms despite initial conservative therapy or simple injection therapies, PNR/PNRBDC could be viable options for the management. Our results are therefore encouraging, showing significant symptomatic, functional, and psychological improvements associated with PNR with and without balloon decompression neuroplasty. In addition, benefits were observed in patients with long-standing symptoms, such as chronic low back pain and lower limb pain from discopathy lasting for more than 3 years, who may still benefit from PNR. Similar conclusions were made in the study of Kim et al. reporting percutaneous adhesiolysis in the general population [27].

### Limitations

Limitations of our study include a low number of cases, the retrospective design, and the irregular follow-up intervals and absence of a control group.

Another limitation is the difference in baseline clinical severity between the two groups (PNR and PNRBDC). Certain parameters were significantly higher in the PNR group at baseline compared to the PNRBDC group.

## 5. Conclusions

Further studies and observations are required to guide the management of LSDS in athletes. While we acknowledge the aforementioned limitations of the study, both interventions, PNR and PNRBDC, were associated with meaningful benefit for the athletes affected by LSDS as observed in our cohort. These minimally invasive techniques were associated with relatively fast RTS, limited disabling status, and prompted relief of pain in our patients’ cohort. The duration of symptoms before performing these interventions did not adversely affect the outcome. We believe, however, that earlier referral should be considered to avoid unnecessary loss of athletic performance and delayed return to sport, when conservative therapy proves ineffective in allowing RTS.

## Figures and Tables

**Table 1:** Characteristics of the patients at baseline (*n* = 33).

Variables	Perineural Release (PNR) *n* = 21	PNR Balloon Decompression Neuroplasty (PNRBDC) *n* = 12
Age (Mean ± SD)	29.5 ± 7.2	29.1 ± 8.0
Gender		
Female	4 (19.0)	1 (8.3)
Male	17 (81.0)	1 (91.7)
Previous minor interventions		
No	17 (81.0)	9 (75.0)
Yes	4 (19.0)	3 (25.0)
Duration of symptoms before PNR in months (Mean ± SD); Median [IQR]	21.1 ± 23.6 24 [3–24]	20.8 ± 26.5 6 [1.5–36.0]
Duration between intervention and follow-up assessment (days)		
≤30	8 (38.1)	2 (16.7)
30–90	8 (38.1)	5 (41.7)
90–120	1 (4.8)	1 (8.3)
120–360	2 (9.5)	1 (8.3)
360+	2 (9.5)	3 (25.0)
EQ-5D		
Mobility	3.2 ± 1.2	2.4 ± 1.0
Pain	4.0 ± 0.5 *	3.3 ± 0.9
Self-care	2.8 ± 1.3	2.3 ± 1.1
Active daily living	3.6 ± 0.9 *	2.8 ± 0.8
Anxiety/depression	3.4 ± 0.8	2.8 ± 1.2
Quality of sleep	2.7 ± 1.2 *	1.8 ± 1.0

* Significantly higher in PNR compared to PNRBDC.

**Table 2:** Correlation tests of level of change in PROMS vs. duration of follow-up.

Outcome			Follow-up_Duration-Month	Change in Mobility	Change in Pain	Change in Self-Care	Change in Usual Activities	Change in Anxiety–Depression	Change in Sleep
Spearman’s rho	Follow-up_Duration-Month	Correlation Coefficient	1.000	−0.404	0.233	−0.296	0.221	−0.157	−0.143
		Sig (2-tailed)		0.020	0.213	0.094	0.216	0.382	0.428
		N	33	33	33	33	33	33	33

**Table 3:** EQ-5D, pain and duration of pain-free activity pre- and post-perineural release (PNR) and PNR balloon decompression neuroplasty (*n* = 33).

	Pre		Post		Cohen’s d	*p*-Value
Variable	Mean ± SD	Median [IQR]	Mean ± SD	Median [IQR]		
EQ-5D						
Mobility	2.9 ± 1.2	3 [2–4]	1.5 ± 0.8	1 [1–2]	1.0	<0.001
Active daily living	3.3 ± 1.0	3 [3–4]	1.6 ± 0.8	1 [1–2]	1.5	<0.001
Self-care	2.6 ± 1.2	3 [1–4]	1.3 ± 0.7	1 [1–1]	1.1	<0.001
Pain	3.8 ± 0.8	4 [3–4]	2.0 ± 0.7	2 [2–2]	1.7	<0.001
Anxiety/depression	3.2 ± 1.0	3 [2–4]	1.4 ± 0.7	1 [1–2]	1.6	<0.001
Quality of sleep	2.3 ± 1.2	3 [1–3]	1.1 ± 0.3	1 [1–1]	1.1	<0.001
Pain						
Max. intensity of pain	7.5 ± 1.7	8 [6–8]	2.8 ± 2.2	3 [1–4]	1.9	<0.001
Min. intensity of pain	3.8 ± 2.3	4 [2–6]	0.3 ± 0.7	0 [0–0.5]	1.5	<0.001
Average intensity of pain	5.6 ± 1.7	6 [4.5–6.5]	1.5 ± 1.2	1.5 [0.5–2.5]	2.0	<0.001
Duration of pain-free activity (minutes)						
Maximum time patient is able to walk without pain (minutes)	24.8 ± 23.2	15 [5–60]	49.7 ± 26.7	60 [30–60]	0.9	<0.001
Maximum standing time (without pain)	18.4 ± 20.0	10 [5–15]	44.1 ± 23.1	60 [20–60]	1.1	<0.001
Maximum sitting time (without pain)	25.5 ± 23.7	15 [5–60]	44.6 ± 24.9	60 [30–60]	0.7	<0.001
Self-reported patient health score (0–100)	51.9 ± 22.6	55 [30–70]	83.0 ± 14.3	90 [77.5–90]	1.2	<0.001

**Table 4:** EQ-5D, pain and duration of pain-free activity pre- and post-perineural release (PNR) (*n* = 21).

**Variable**	**Pre**		**Post**		**Cohen’s d**	***p*-Value**
	**Mean ± SD**	**Median [IQR]**	**Mean ± SD**	**Median [IQR]**		
Mobility	3.2 ± 1.2	4 [2–4]	1.5 ± 0.8	1 [1–2]	1.2	<0.001
Active daily living	3.6 ± 0.9	4 [3–4]	1.5 ± 0.7	1 [1–2]	2.0	<0.001
Self-care	2.8 ± 1.3	3 [2–4]	1.3 ± 0.7	1 [1–1]	1.2	<0.001
Pain	4.0 ± 0.5	4 [4–4]	2.0 ± 0.7	2 [2–2]	2.4	<0.001
Anxiety/depression	3.4 ± 0.8	3 [3–4]	1.3 ± 0.6	1 [1–2]	2.5	<0.001
Quality of sleep	2.7 ± 1.2	3 [1–4]	1.1 ± 0.4	1 [1–1]	1.3	<0.001
Max. intensity of pain	8.0 ± 1.4	8 [8–9]	2.7 ± 2.2	3 [0.5–4]	2.5	<0.001
Min. intensity of pain	4.4 ± 2.0	4 [3–6]	0.4 ± 0.7	0 [0–0.5]	1.9	<0.001
Average intensity of pain	6.2 ± 1.4	6 [5–7]	1.5 ± 1.2	2 [0–2.5]	2.8	<0.001
Maximum time patient is able to walk without pain (minutes)	21.5 ± 20.9	15 [5–30]	51.0 ± 15.5	60 [40–60]	1.2	<0.001
Maximum standing time (without pain)	14.3 ± 17.1	5 [5–15]	44.0 ± 19.7	60 [30–60]	1.4	<0.001
Maximum sitting time (without pain)	19.3 ± 21.8	10 [5–30]	46.7 ± 26.2	60 [30–60]	1.1	<0.001
Self-reported patient health score (0–100)	49.3 ± 23.0	55 [30–65]	84.8 ± 14.4	90 [77.5–92.5]	1.4	<0.001

**Table 5:** EQ-5D, pain and duration of pain-free activity pre- and post-PNR balloon decompression neuroplasty (*n* = 12).

Variable	Mean ± SD	Median [IQR]	Mean ± SD	Median [IQR]	Cohen’s d	*p*-Value
Mobility	2.4 ± 1.0	2 [2–3]	1.7 ± 0.8	1.5 [1–2]	0.8	0.030
Active daily living	2.8 ± 0.8	3 [2.5–3]	1.7 ± 0.8	1.5 [1–2]	1.0	0.012
Self-care	2.3 ± 1.1	2.5 [1–3]	1.3 ± 0.7	1 [1–1.5]	0.8	0.021
Pain	3.3 ± 0.9	3 [3–4]	2.0 ± 0.7	2 [2–2]	1.0	0.006
Anxiety/depression	2.8 ± 1.2	2.5 [2–4]	1.6 ± 0.9	1 [1–2.5]	0.9	0.012
Quality of sleep	1.8 ± 1.0	1 [1–3]	1.0 ± 0.0	1 [1–1]	0.8	0.034
Max. intensity of pain	6.5 ± 1.8	7 [5.5–8]	2.9 ± 2.3	2.5 [1.5–4]	1.3	0.004
Min. intensity of pain	2.7 ± 2.5	2 [0–4.5]	0.3 ± 0.7	0 [0–0.5]	1.0	0.011
Average intensity of pain	4.6 ± 1.7	4.5 [3–5.75]	1.6 ± 1.3	1.5 [1–2]	1.4	0.002
Maximum time patient is able to walk without pain (minutes)	30.8 ± 26.7	20 [5–60]	47.5 ± 40.4	45 [15–60]	0.6	0.058
Maximum standing time (without pain)	25.4 ± 23.3	12.5 [10–52.5]	44.2 ± 29.1	60 [17.5–60]	0.7	0.011
Maximum sitting time (without pain)	36.3 ± 23.9	37.5 [10–60]	40.8 ± 23.0	55 [20–60]	0.2	0.344
Self-reported patient health score (0–100)	56.3 ± 22.3	55 [35–70]	80.0 ± 14.3	80 [75–90]	0.9	0.026

**Table 6:** Distribution of EQ-5D health status domains pre- and post-PNR procedures (*n* = 33).

EQ-5D	Status	None–Mild (%)	Moderate–Severe–Unable (%)
Mobility	Pre	14 (42.4%)	19 (57.6%)
	Post	29 (87.9%)	4 (12.1%)
ADLs	Pre	5 (15.2%)	28 (84.8%)
	Post	28 (84.8%)	5 (15.2%)
Self-Care	Pre	15 (45.5%)	18 (54.5%)
	Post	29 (87.9%)	4 (12.1%)
Pain and Discomfort	Pre	2 (6.1%)	31 (93.9%)
	Post	27 (81.8%)	6 (18.2%)
Anxiety/Depression	Pre	9 (27.3%)	24 (72.7%)
	Post	29 (87.9%)	4 (12.1%)
Quality of Sleep	Pre	15 (45.5%)	18 (54.5%)
	Post	32 (97.0%)	1 (3.0%)

## Data Availability

Data related to this study is available from the corresponding author upon request.
